# No evidence for seasonal variations of the incidence of testicular germ cell tumours in Germany

**DOI:** 10.1371/journal.pone.0286309

**Published:** 2023-05-26

**Authors:** Klaus-Peter Dieckmann, Hendrik Isbarn, Pietro Trocchi, Marvin Kießling, Christian Wülfing, Andreas Stang

**Affiliations:** 1 Hodentumorzentrum, Urologische Abteilung, Asklepios Klinik Altona, Hamburg, Germany; 2 Martini Klinik, Universitätsklinikum Eppendorf, Hamburg, Germany; 3 Institut für Medizinische Informatik, Biometrie und Epidemiologie, Universitätsklinikum Essen, Essen, Germany; 4 Cancer Registry of North Rhine-Westphalia, Bochum, Germany; University of Bern: Universitat Bern, SWITZERLAND

## Abstract

The pathogenesis of testicular germ cell tumours (GCTs) is still incompletely understood. Any progress in its understanding must derive from observational studies. Recently, it has been suggested that the incidence of GCTs may follow a seasonal pattern based on circannual changes in the Vitamin D serum levels, with maximum incidence rates in winter months. To examine this promising hypothesis, we studied monthly incidence rates of testicular GCTs in Germany by analysing 30,988 GCT cases aged 15–69 years, diagnosed during 2009–2019. Monthly incident case numbers with data regarding histology and patient age were obtained from the Robert Koch Institut, Berlin, along with annual male population counts. We used precision weighting for deriving pooled monthly incidence rates for GCTs of the period 2009–2019. We stratified pooled rates by histology (seminoma and nonseminoma) and age (15–39 and 40–69 years). By assuming a cyclical effect, we used an estimator of the intensity of seasonal occurrence and report seasonal relative risks (RR). The mean monthly incidence rate was 11.93/10^5^ person-months. The seasonal RR for testicular cancer over-all is 1.022 (95% CI 1.000–1.054). The highest seasonal RR was found in the subgroup of nonseminoma aged 15–39 years, with a RR 1.044 (95% CI 1.000–1.112). The comparison of the pooled monthly rates of the winter months (October—March) with the summer months (April-September) revealed a maximum relative difference of 5% (95% CI 1–10%) for nonseminoma, aged 15–39 years. We conclude that there is no evidence of a seasonal variation of incidence rates of testicular cancer. Our results are at odds with an Austrian study, but the present data appear sound because the results were obtained with precision weighted monthly incidence rates in a large population of GCT cases.

## Introduction

There is wide-spread international consensus that adult testicular germ-cell tumours (GCTs) derive from germ cell neoplasia in situ (GCNis). These precursor cells origin from primordial germ cells that fail to follow the normal maturation process of embryonic germ cells during embryogenesis [1]. As these cells keep their embryonic pluripotency characteristics during later life, they may develop to germ cell neoplasms after puberty [2, 3]. While the basic principles of this theory are undisputed, the details of the pathogenetic pathway are widely unknown [4]. As there is no experimental model of GCT and as animal testicular tumours are different from their human counterparts [5], any progress in understanding the pathogenesis of human GCTs must rely on systematic observation studies in all fields of clinical and preclinical medicine. Particularly, epidemiological studies involve a great potential of generating hypotheses. Recently, a study by the National Austrian Cancer Registry reported a significant seasonal variation in the incidence of GCT with peak incidence rates in winter months, October to December, and January to March [6]. The authors suggested sun-exposure related seasonal variations of Vitamin D3 serum levels to be associated with the changes in the GCT incidence rates. This hypothesis appears quite appealing because—if confirmed in a larger patient population—it could also be a clue for other epidemiological peculiarities of GCTs such as the north-south gradient of incidence [7]. Also, as GCTs afflict the male reproductive organs, seasonal variation of the GCT incidence would be consistent with the recently documented marked circannual variations of sperm parameters [8]. Therefore, we studied the monthly incidence rates of adult GCTs arising in Germany during the last decade.

## Material & methods

In Germany, there is no national cancer registry, but reporting incident cancer cases to the cancer registries of the federal states is compulsory for all cancer-care providing institutions. The Centre for Cancer Registry Data (Zentrum für Krebsregisterdaten, ZfKD) a subdivision of the Robert Koch-Institut (RKI), Berlin, routinely collects records from all population-based cancer registries in Germany. After quality control of the incoming records, the data are merged into a central national database annually.

We received data from the ZfKD on all incident cases of primary malignant testicular cancer (ICD-10) aged 15–69 years and diagnosed 2009–2019. We included only data from the federal states of Niedersachsen, Schleswig-Holstein, Hamburg, Bremen, Nordrhein-Westfalen, Saarland, Hessen, Rheinland-Pfalz, Baden-Württemberg, and Bayern, because these states have an estimated completeness of registration above 90% in each year of the period of 2009–2019. Federal states reporting data only for parts of the entire observation period were excluded from the present analysis. The number of cases identified by death certificate only (DCO) was 2%. In addition, we received the official German population count numbers for each of the calendar years 2009–2019 from the RKI by age groups [9]. As no monthly populations counts were available, we assumed an even distribution over the year and calculated the assumed monthly population counts by simply dividing the annual count by 12.

As the focus of the present study was specifically on testicular germ-cell tumours, we used ICD-O morphology codes to categorize testicular neoplasms as seminoma (ICD-O-3: 9061–9063), nonseminoma (ICD-O-3: 9065-9085/3), other testicular cancers (dysgerminoma [9060/3] and germinoma not otherwise specified [9064/3]).

We did not differentiate spermatocytic tumour cases from classical seminoma because this entity is clinically very similar to seminoma, it is very rare (< 1% of all seminoma) and it is characterized by frequent patho-histological mis-classifications [10].

Ethical approval was provided by Ethical committee of Ärztekammer Hamburg on May 20, 2022 (2022-100828-BO-ff). The research was carried out at the Asklepios Klinik Altona, Hamburg, Germany, and in the Institut für Medizinische Informatik, Biometrie und Epidemiologie, Universitätsklinikum Essen, Germany. The need for informed consent of patients was waived by the ethical committee since only registry-based anonymous data were used for analysis.

### Statistical methods

We first calculated the annual incidence of the entire population of testicular cancer for the observation period 2009–2019 (cases/10^5^ person-years [py]). We then calculated monthly incidence rates for each year, and stratified by histologic group (seminoma and nonseminoma) and age (15–39 years, and 40–69 years). Stratification by age categories was done because the clinical features of younger patients (<40 years) are somewhat different from those of older patients and it thus appeared rational to look for differences regarding epidemiological characteristics. Another reason for analysing age categories was to be consistent with the Austrian report [6].

We weighted each monthly rate by its precision, that is, by the inverse of its variance and thereafter pooled the monthly rates across the 11 years month by month [11]. We used inverse-variance weighting for pooling monthly incidence rates of each of the 11 years. For each pooled incidence rate, we calculated 95% confidence intervals (CIs). Monthly incidence rates are reported as cases per 10^5^ person-months (pm).

For the graphical display of pooled monthly rates, we also calculated the average of monthly incidences across all pooled monthly rates to make deviations from the annual average of the rates easily visible.

To estimate the intensity of seasonal occurrence of GCT, we used an estimator of the intensity of seasonal occurrence. This estimator is based on the assumption of a single cyclical effect (harmonic) that can be well approximated by a sine curve [12, 13]. We used the EpiSheet workbook to estimate the peak/low ratio [14]. The estimated peak/low ratio is also called the seasonal relative risk (RR).

An additional analysis was done by comparing winter months (October-March) with summer months (April—September). The incidence rates for these two seasons were computed after precision-weighted pooling of the monthly rates (October-March and April-September). Finally, we determined the ratio of the winter and summer rate with calculating 95% CIs.

## Results

A total of 30,988 cases were included in the present analysis, thereof 19,936 (64.3%) cases with seminoma, 10,164 (32.8%) with nonseminoma, 17 (0.05%) with dysgerminoma, not other specified, and 871 (2.8%) with germinoma not other specified. A total of 131 cases with spermatocytic tumour (ICD-O-3: 9063/3; formerly called spermatocytic seminoma) were included in the group of seminoma. Based on the population count numbers 2009–2019, the over-all incidence rate during the complete observation period was 12.02/10^5^py. The incidence rates in the histologic subgroups and in age categories are listed in Table 1.

**Table 1:** Overall annual incidence rates of testicular cancer in Germany, 2009–2019.

Entity	Age categories (years)	Cases (n)	Rate (10^5^ py)	95% CI
C62, overall	15–69	30,988	12.02	11.88–12.15
Seminoma	15–69	19,936	7.73	7.62–7.84
Nonseminoma	15–69	10,164	3.94	3.86–4.02
C62, overall	15–39	17,533	16.05	15.81–16.29
Seminoma	15–39	9,262	8.48	8.31–8.65
Nonseminoma	15–39	7,701	7.05	6.89–7.21
C62, overall	40–69	13,455	9.05	8.90–9.21
Seminoma	40–69	10,674	7.18	7.04–7.32
Nonseminoma	40–69	2,463	1.66	1.59–1.72

SE standard error; py person-years; C62 ICD-10 Code for malignant testicular neoplasm

**Fig 1:**
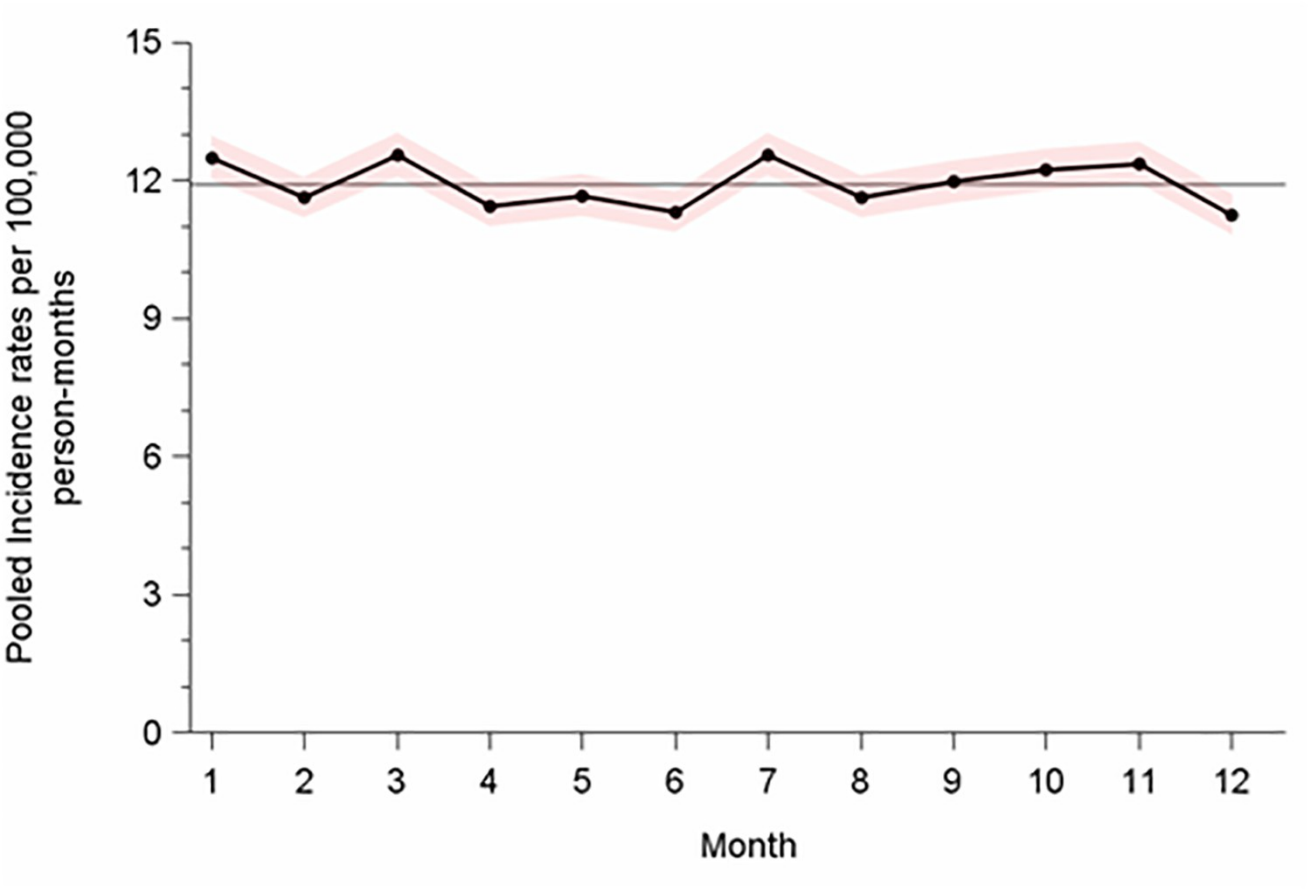
Precision-weighted month-specific rates of testicular cancer among men aged 15–69 years, Germany 2009–2019. Horizontal line indicates the mean rate across months 1–12; shadowed areas denote 95% confidence intervals.

The mean monthly incidence rate across all precision-weighted monthly incidence rates for the entire population of GCT during 2009–2019 was 11.93/10^5^pm. Monthly incidence rates showed barely any variation over the year (Fig 1).

**Fig 2:**
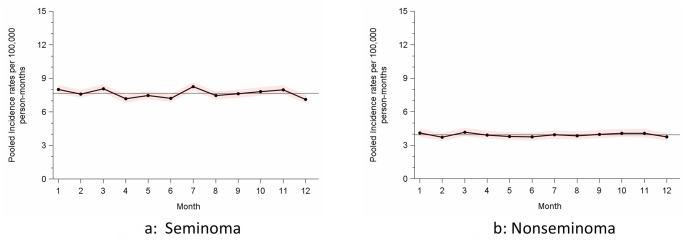
Precision-weighted month-specific rates of GCT patients in Germany, 2009–2019, stratified by histology. Horizontal line indicates the mean rate across months 1–12; shadowed areas denote 95% confidence intervals.

Stratification by histology (Table 2) revealed an over-all monthly incidence of 7.65/10^5^pm and 3.93/10^5^ pm for seminoma and for nonseminoma, respectively. The seasonal RR was 1.022 (95% CI 1.000–1.054). As shown in Fig 2a and 2b, monthly incidence rates showed barely any variation over the year in either histologic group.

**Table 2:** Precision-weighted monthly incidence rates of testicular cancer in Germany, 2009–2019, with stratifications for histology and age categories.

population	Age (years)	Cases overall (n)	minimum rate (per 10^5^ pm)	Mean Rate (per 10^5^ pm)	maximum rate (per 10^5^ pm)	Seasonal relative risk	95%CI
C62 overall	**15–69**	30,988	11.25	11.93	12.57	1.022	1.000–1.054
Seminoma		19,936	7.13	7.65	8.26	1.017	1.000–1.058
Nonseminoma		10,164	3.73	3.93	4.18	1.026	1.000–1.084
C62 overall	**15–39**	17,533	14.55	15.88	16.92	1.043	1.000–1.087
Seminoma		9,262	7.56	8.35	9.24	1.057	1.000–1.120
Nonseminoma		7,701	6.40	6.91	7.56	1.044	1.000–1.112
C62 overall	**40–69**	13,455	8.17	8.94	9.68	1.005	1.000–1.054
Seminoma		10,674	6.42	7.06	7.52	1.016	1.000–1.072
Nonseminoma		2,463	1.41	1.58	1.79	1.028	1.000–1.149

Rates are expressed as cases per 100.000 person-months; C62 ICD-10 Code for malignant testicular neoplasm

**Fig 3:**
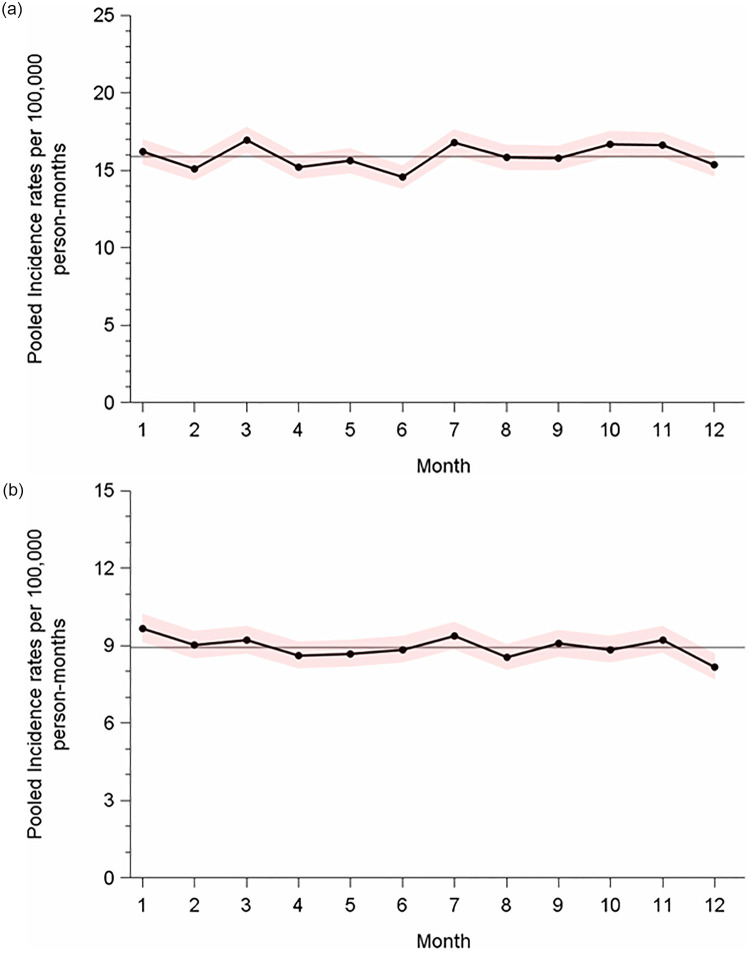
**a.** Precision-weighted monthly incidence rates of testicular cancer in Germany, 2009–2019, stratified by age categories: 15–39 years. **b.** Precision-weighted monthly incidence rates of testicular cancer in Germany, 2009–2019, stratified by age categories: 40–69 years. Horizontal line indicates the mean rate across months 1–12; shadowed areas denote 95% confidence intervals.

Stratification of the monthly incidence rates by age groups (Fig 3a and 3b) revealed over-all incidences of 15.88/10^5^pm and 8.94/10^5^pm in men aged 15–39 years and in those aged 40–69 years, respectively. Monthly deviations from the average were very small in both age categories. Detailed results of the seasonal analyses of all subpopulations are given in Table 2. The seasonal RR is close to 1.0 in all subgroups with a range of 1.005 (minimum) to 1.057 (maximum), indicating that the highest monthly rate was at most 5.7% higher than the lowest monthly rate.

Comparisons of the pooled monthly rates in the winter season (October—March) with the pooled rate in the summer season (April—September) regarding the total population and its subpopulations are listed in Table 3. Consistent with our other findings, there was very little difference of the incidence rates between the two seasons for the over-all group, the histology-specific groups and age -specific groups. This result is exemplified by the incidence rate for the entire population of testicular cancer (C62 overall, age group 15–69 years) which was relatively 3% higher in winter than in summer (12.16 versus 11.84 per 10^5^ pm, rate ratio 1.03, 95%CI 1.00–1.05).

**Table 3:** Comparison of the incidence rate of testicular cancer in the winter (Oct-Mar) and summer (Apr-Sep) months in Germany, 2009–2019.

	Cases (n)		Incidence rates (per 10^5^ pm) & 95%CI				Rate Ratio & 95%CI*	
	Winter	Summer	Winter		Summer		Rate Ratio	95%CI
**C62**			Rate	95%CI	Rate	95%CI		
15–69	15700	15288	12.16	11.97–12.35	11.84	11.65–12.03	1.03	1.00–1.05
15–39	8919	8614	16.30	15.96–16.64	15.74	15.41–16.07	1.04	1.01–1.07
40–69	6781	6674	9.10	8.88–9.31	8.97	8.75–9.19	1.01	0.98–1.05
**Seminoma**								
15–69	10108	9828	7.83	7.67–7.98	7.61	7.46–7.76	1.03	1.00–1.06
15–39	4692	4570	8.57	8.33–8.82	8.33	8.09–8.58	1.03	0.99–1.07
40–69	5416	5258	7.26	7.07–7.46	7.07	6.87–7.26	1.03	0.99–1.07
**Nonseminoma**								
15–69	5158	5006	3.99	3.88–4.10	3.88	3.77–3.99	1.03	0.99–1.07
15–39	3949	3752	7.22	6.99–7.44	6.86	6.64–7.08	1.05	1.01–1.10
40–69	1209	1254	1.62	1.52–1.71	1.68	1.59–1.77	0.96	0.89–1.04

*Rate from winter divided by rate from summer

## Discussion

There was barely any seasonal variation of the incidence of GCTs in Germany for testicular cancer over-all, the two major histologic subgroups and for age groups. Furthermore, there was practically no difference between the incidence rates in the winter and summer periods. The results of the present study appear methodologically sound, since they were derived from a thorough statistical analysis of a rather large population of GCT patients using precision-weighted monthly incidence rates. The over-all annual incidence rate of GCT in Germany [15], the higher incidence of seminoma compared to that of nonseminoma [16, 17], and the much higher incidence of GCT in younger than in older ages are in line with previous publications [7, 17, 18].

However, our data are at odds with the results of the Austrian group who reported significant increases of the GCT incidence in autumn and winter months [6]. Yet, a closer look at the data of the Austrian study reveals that the difference between the summer and winter period was restricted to the histologic subgroup of seminoma and particularly to cases with localized disease. Among the nonseminomas, no variation of month-specific incidence rates were found. In the present study, no such variations were observed. On the other hand, it is well documented that in seminoma there is usually a very long diagnostic delay, which also applies to localized stages [19–21]. Accordingly, in patients with seminoma, the time-points of establishing the clinical diagnosis and of perceiving first symptoms by the patient are markedly apart which implies that there is likewise a long time interval between the time points of clinical detection and the biologic onset of the disease. These long symptomatic intervals may relate to the usually rather slow growth rate of seminoma if compared to the more aggressive course of nonseminoma [22]. In light of the exceptionally long lag time between biologic onset of disease and clinical diagnosis of seminoma, the seasonal variation of the incidence of seminoma found by the Austrian group is likely to reflect effects unrelated to the disease, such as patient-related variations of bodily self-perception or health-care system associated temporal changes of diagnostic capacities. The hypothesized association of GCT pathogenesis with vitamin D serum levels is thus likewise only little substantiated by our data.

No other epidemiological study has so far analysed seasonal variations of the incidence of testicular cancer. However, indirect support for the present results comes from a study of the Swedish National Cancer Registry that investigated seasonal variations of all cancers in that country [23]. Actually, seasonal variations were found only in the four malignancies of melanoma, and of cancers of breast, prostate, and thyroid. As all other cancers did not exhibit seasonal variations of incidences, it must be assumed—although not unequivocally specified—that there was no seasonal effect in testicular cancer.

Curiously, a seasonal pattern had been reported regarding the months of birth of patients with testicular GCT in UK and in Hungary and the finding was suggested to be related to prenatal infections [24, 25]. However, other studies noted this effect only in selected histologic subgroups, or only in patients succumbing to the disease or solely in particular geographic regions [26–28]. Accordingly, no further consideration had been credited to the birth date hypothesis in recent major reviews on the aetiology and pathogenesis of testicular cancer [1, 22, 29, 30].

In a small number of malignant diseases, seasonal variations of the incidences have been documented. In acute myeloic leukemia, that association was suggested to relate to seasonally changing environmental factors or to infectious agents [31, 32]. Malignant melanoma is more frequently diagnosed in summer months than in winter time [33] and this finding conceivably relates to melanoma-promoting sun exposure but also to easier detection due to light summer clothing [23]. Breast cancer has also repeatedly been found to occur more frequently in winter months [34], but this finding has been linked to mammography screening programmes that are usually less frequently attended in summer months [23]. Finally, thyroid cancer and prostatic cancer have been reported to be diagnosed with circannual rhythms and in these diseases the changing frequencies of clinical diagnoses have been linked to health system-associated temporal changes of diagnostic capacities [23].

In spite of the large sample size, there are several limitations that need to be borne in mind in interpreting the present results. Histological subtyping of testicular cancer was based on the coding of the cancer registries without a central pathologic review. Some histologic misclassification is expected since GCT is a rare disease and less-experienced local pathologists may sometimes fail a correct classification of testicular neoplasms [35]. However, the main result of the present study relating to the over-all analysis without histologic stratification, is probably not affected by this issue. The cases with spermatocytic tumour were included in the subgroup of seminoma although it is pathogenetically different from seminoma according to the most recent patho-histological classification system [36]. Cases registered with death certificate only (DCO) were included in the analysis with their date of death as a surrogate of the date of diagnosis of testicular cancer if not otherwise specified on the death certificate. However, we believe that both, spermatocytic tumour cases and DCO cases had no major impact on the over-all results of this study because the basic findings are very clear-cut, and because both groups involve very small numbers in relation to the over-all large sample size. A minor limitation of the study might result from the lack of monthly population counts and from the assumption of an even distribution of population counts over the respective years. The present evaluation comprised of cases from Germany only and mostly included Caucasians. Thus, it is unclear whether our results can be generalized to other ethnicities.

In conclusion, we found barely any seasonal variation of the incidence of testicular cancer. Our results are in conflict with a recent Austrian study [6]. However, as the present evaluation involves an almost ten-fold larger patient population than the Austrian study, the weight of evidence of the present investigation appears greater. In conjunction with a Swedish cancer registry study that indirectly reported a null finding, too, there is apparently no evidence for a seasonal variation of the incidence of testicular GCT.

## References

[1] Rajpert-De Meyts E, McGlynn KA, Okamoto K, Jewett MA, Bokemeyer C. Testicular germ cell tumours. Lancet. 2016;387(10029):1762–74. doi: 10.1016/S0140-6736(15)00991-5 26651223

[2] Skakkebaek NE, Berthelsen JG, Giwercman A, Müller J. Carcinoma in situ of the testis: possible origin from gonocytes and precursor of all types of germ cell tumours except spermatocytoma. Int J Androl. 1987;10:19–28. doi: 10.1111/j.1365-2605.1987.tb00161.x 3034791

[3] Dieckmann KP, Skakkebaek NE. Carcinoma in situ of the testis: A review of biological and clinical features. Int J Cancer. 1999;83:815–22. 10597201 10.1002/(sici)1097-0215(19991210)83:6<815::aid-ijc21>3.0.co;2-z

[4] Hoei-Hansen CE, Rajpert-De Meyts E, Daugaard G, Skakkebaek NE. Carcinoma in situ testis, the progenitor of testicular germ cell tumours: a clinical review. Ann Oncol. 2005;16:863–8. doi: 10.1093/annonc/mdi175 15821122

[5] Bush JM, Gardiner DW, Palmer JS, Rajpert-De Meyts E, Veeramachaneni DN. Testicular germ cell tumours in dogs are predominantly of spermatocytic seminoma type and are frequently associated with somatic cell tumours. Int J Androl. 2011;34(4 (pt2)):e288–e95. doi: 10.1111/j.1365-2605.2011.01166.x 21615421

[6] Tulchiner G, Staudacher N, Fritz J, Hackl M, Pichler M, Seles M, et al. Seasonal Variations in the Diagnosis of Testicular Germ Cell Tumors: A National Cancer Registry Study in Austria. Cancers (Basel). 2021;13(21):5377. doi: 10.3390/cancers13215377 34771540 PMC8582382

[7] Znaor A, Skakkebaek NE, Rajpert-De Meyts E, Kuliš T, Laversanne M, Gurney J, et al. Global patterns in testicular cancer incidence and mortality in 2020. Int J Cancer. 2022;151(5):692–8. doi: 10.1002/ijc.33999 35277970 PMC12980092

[8] Santi D, Magnani E, Michelangeli M, Grassi R, Vecchi B, Pedroni G, et al. Seasonal variation of semen parameters correlates with environmental temperature and air pollution: A big data analysis over 6 years. Environ Pollut. 2018;235:806–13. doi: 10.1016/j.envpol.2018.01.021 29353799

[9] Robert Koch Institut, Zentrum für Krebsregisterdaten (ZfKD). Datensatz des ZfKD auf Basis der epidemiologischen Landeskrebsregisterdaten, verfügbare Diagnosejahre bis 2019. Version: Epi2022_12022.

[10] Sharma P, Dhillon J, Agarwal G, Zargar-Shoshtari K, Sexton WJ. Disparities in Interpretation of Primary Testicular Germ Cell Tumor Pathology. Am J Clin Pathol. 2015;144(2):289–94. doi: 10.1309/AJCPJTX8R6CVWSRW 26185314

[11] Sutton AJ, Abrams KR, Jones DR, Sheldon T, Song F. Methods for meta-analysis in medical research. Chichester: John Wiley; 2000. 58 p.

[12] Edwards JH. The recognition and estimation of cyclic trends. Ann Hum Genet. 1961;25:83–7; doi: 10.1111/j.1469-1809.1961.tb01501.x 13725808

[13] Brookhart MA, Rothman KJ. Simple estimators of the intensity of seasonal occurrence. BMC Med Res Methodol. 2008;8(1):67; doi: 10.1186/1471-2288-8-67 18945366 PMC2596789

[14] www.rtihs.org. 2022 [accessed Juli 10, 2022].

[15] Bertz J, Buttmann-Schweiger N, Kraywinkel K. Epidemiologie bösartiger Hodentumoren in Deutschland. Onkologe. 2017;23(2):90–6.

[16] Ruf CG, Isbarn H, Wagner W, Fisch M, Matthies C, Dieckmann KP. Changes in epidemiologic features of testicular germ cell cancer: Age at diagnosis and relative frequency of seminoma are constantly and significantly increasing. Urol Oncol. 2014;32(1):33.e1–6. doi: 10.1016/j.urolonc.2012.12.002 23395239

[17] Stang A, Rusner C, Eisinger B, Stegmaier C, Kaatsch P. Subtype-specific incidence of testicular cancer in Germany: a pooled analysis of nine population-based cancer registries. Int J Androl. 2009;32(4):306–16. doi: 10.1111/j.1365-2605.2007.00850.x 18179558

[18] Stang A, Rusner C, Stabenow R. Changing epidemiologic features of testicular germ cell cancer in Germany: corroboration at population level. Urol Oncol. 2013;31(8):1839–40. doi: 10.1016/j.urolonc.2013.07.007 24035419

[19] Dieckmann KP, Becker T, Bauer HW. Testicular Tumors: Presentation and Role of Diagnostic Delay. Urol Int. 1987;42:241–7. doi: 10.1159/000281948 3314057

[20] Huyghe E, Muller A, Mieusset R, Bujan L, Bachaud JM, Chevreau C, et al. Impact of diagnostic delay in testis cancer: Results of a large population based study. Eur Urol. 2007;52:1710–6. doi: 10.1016/j.eururo.2007.06.003 17618044

[21] Moul JW. Timely diagnosis of testicular cancer. Urol Clin North Am. 2007;34:109–17. doi: 10.1016/j.ucl.2007.02.003 17484916

[22] Cheng L, Albers P, Berney DM, Feldman DR, Daugaard G, Gilligan T, et al. Testicular cancer. Nat Rev Dis Primers. 2018;4(1):29. doi: 10.1038/s41572-018-0029-0 30291251

[23] Lambe M, Blomqvist P, Bellocco R. Seasonal variation in the diagnosis of cancer: a study based on national cancer registration in Sweden. Br J Cancer. 2003;88(9):1358–60. doi: 10.1038/sj.bjc.6600901 12778061 PMC2741038

[24] Knox EG, Cummins C. Birth dates of men with cancer of the testis. J Epidemiol Community Health. 1985;39(3):237–43. doi: 10.1136/jech.39.3.237 4045366 PMC1052442

[25] Klujber V, Baki M, Bodogri I. Epidemiology of germinal cell testis cancer in Hungary. (in Hungarian). Orvosi hetilap. 1990;131:975–8. 2189094

[26] Bernstein L, Chilvers C, Murrells T, Pike MC. Month of birth of men with malignant germ cell tumours of the testis. J Epidemiol Community Health. 1986;40(3):214–7. doi: 10.1136/jech.40.3.214 3021889 PMC1052525

[27] Kinlen LJ, Willows AN. Cancer of the testis and month of birth. Br J Cancer. 1987;55(5):579–81. doi: 10.1038/bjc.1987.117 3606950 PMC2001734

[28] McNally RJ, Pearce MS, Parker L. Space-time clustering analyses of testicular cancer amongst 15-24-year-olds in Northern England. Eur J Epidemiol. 2006;21(2):139–44. doi: 10.1007/s10654-005-5698-9 16518682

[29] De Toni L, Šabovic I, Cosci I, Ghezzi M, Foresta C, Garolla A. Testicular Cancer: Genes, Environment, Hormones. Front Endocrinol (Lausanne). 2019;10:408, doi: 10.3389/fendo.2019.00408 31338064 PMC6626920

[30] Huang J, Chan SC, Tin MS, Liu X, Lok VT, Ngai CH, et al. Worldwide Distribution, Risk Factors, and Temporal Trends of Testicular Cancer Incidence and Mortality: A Global Analysis. Eur Urol Oncol. 2022; 5 (5):566–76. doi: 10.1016/j.euo.2022.06.009 35863988

[31] Calip GS, McDougall JA, Wheldon MC, Li CI, De Roos AJ. Evaluation of seasonality in the diagnosis of acute myeloid leukaemia among adults in the United States, 1992–2008. Br J Haematol. 2013;160(3):343–50 doi: 10.1111/bjh.12137 23189956 PMC3552069

[32] Sánchez-Vizcaíno F, Tamayo C, Ramos F, Láinez-González D, Serrano-López J, Barba R, et al. Identification of seasonal variation in the diagnosis of acute myeloid leukaemia: a population-based study. Br J Haematol. 2022;198: (3): 545–55. doi: 10.1111/bjh.18279 35639095 PMC9542150

[33] Braun MM, Tucker MA, Devesa SS, Hoover RN. Seasonal variation in frequency of diagnosis of cutaneous malignant melanoma. Melanoma Res. 1994;4(4):235–41. doi: 10.1097/00008390-199408000-00005 7950359

[34] Cohen P, Wax Y, Modan B. Seasonality in the occurrence of breast cancer. Cancer Res. 1983;43:892–6. 6848200

[35] Harari SE, Sassoon DJ, Priemer DS, Jacob JM, Eble JN, Caliò A, et al. Testicular cancer: The usage of central review for pathology diagnosis of orchiectomy specimens. Urol Oncol. 2017;35:605.e9–e16. doi: 10.1016/j.urolonc.2017.05.018 28647396

[36] Berney DM, Cree I, Rao V, Moch H, Srigley JR, Tsuzuki T, et al. An Introduction to the WHO 5th Edition 2022 Classification of Testicular tumours. Histopathology. 2022; 81 (4): 459–66. doi: 10.1111/his.14675 35502823 PMC9544657

